# Mechanics of compression-driven morphogenesis

**DOI:** 10.1242/dev.205781

**Published:** 2026-08-24

**Authors:** Jue Yu Kelly Tan, Chii Jou Chan

**Affiliations:** ^1^ Mechanobiology Institute, National University of Singapore, Singapore 117411; ^2^ Department of Biological Sciences, National University of Singapore, Singapore 117558

**Keywords:** Tissue pressure, Compressive stress, Tissue mechanics, Mechanotransduction, Morphogenesis

## Abstract

Mechanical forces shape multiscale biological processes across many living systems, ranging from cell proliferation and apoptosis, to tissue folding and pattern formation. In recent years, tissue pressure has emerged as a fundamental regulator of morphogenesis across animal and plant kingdoms. However, a fundamental understanding of the definition and origin of tissue pressure remains lacking. In this Review, we synthesise how tissue pressure is generated from diverse sources, including tissue growth, actomyosin contractility, topological defects and fluid- or extracellular matrix-driven swelling, and how it influences developmental processes such as proliferation, apoptosis, differentiation, tissue folding and pattern formation. We also discuss how homeostatic pressure governs tissue growth and competition, while highlighting the cellular and molecular signalling pathways implicated in compressive stress responses. Finally, drawing on examples from animal, plant, bacterial and organoid systems, we outline emerging experimental and computational approaches to measure and manipulate tissue pressure *in vivo*, and identify key challenges in dissecting compression-specific mechanosignalling pathways.

## Teaser

**Summary:** We synthesise how tissue pressure is generated from diverse sources, and how it influences developmental processes such as proliferation, apoptosis, differentiation, tissue folding and pattern formation.

## INTRODUCTION

Morphogenesis is a highly programmed process that transforms cells into highly organised tissues with reproducible size and structure. Traditionally, genetic and biochemical signalling pathways have been viewed as the primary drivers of morphogenesis. However, accumulating evidence has made it increasingly clear that mechanical forces play indispensable roles governing development across animals and plants (Collinet and Lecuit, 2021; Harmansa and Lecuit, 2021; Heisenberg and Bellaïche, 2013). Cells continuously generate, transmit and respond to various mechanical stresses (see Glossary), which can be propagated across tissues to influence cell proliferation, apoptosis and differentiation. Among these mechanical signals, tissue pressure – defined as the isotropic component of mechanical stress arising from multiple factors such as cell contractility, fluid accumulation and geometric constraints – has emerged as a central regulator of developmental processes across many kingdoms due to its ability to propagate over long distances (Delarue, 2025). Tissue pressure can be transmitted through interconnected cell–matrix networks and fluids across entire tissues or organs. This allows the coordination of cellular behaviours across multiple scales, linking molecular processes such as mechanotransduction to emergent tissue-level phenomena, including growth, patterning and morphogenesis.

**Table:** 

**Glossary**
**Compressive stress**
The directional (tensorial) component of mechanical stress that acts to squeeze a material, arising from forces acting normal to a surface. Compressive stress can be anisotropic (directional) or isotropic (uniform), and it corresponds to pressure (a scalar) when it is completely isotropic.
**Hydrostatic pressure**
The pressure exerted by a fluid at equilibrium. In biological tissues, hydrostatic pressure can result from cell-mediated fluid secretion or ion transport that sets up an osmotic gradient.
**Mechanical stress**
The internal distribution of forces that biological tissues exert on neighbouring regions to resist deformation. When a tissue region experiences a force from its surroundings, mechanical stress is defined as the magnitude of that force divided by the area of contact
**Nematic material**
This structure consists of elongated elements (such as cells or fibres) that exhibit collective orientational order with no positional order.
**Osmotic pressure**
Pressure exerted by fluid when there is an imbalance in solute concentrations across a semi-permeable membrane. Osmotic pressure differences can thus cause cells or tissues to swell in hypotonic environments or shrink in hypertonic environments.
**Poroelastic materials**
Such materials consist of an elastic solid matrix interlinked with fluid-filled pores. They exhibit two-way coupling: fluid pressure in the pores of the matrix applies internal stress that strains the porous matrix, while deformation of the porous solid matrix drives inward or outward flow of the fluid.
**Pressure homeostasis**
Dynamic feedback process to maintain tissue pressure within an optimal physiological range by balancing stress-generating (e.g. proliferation and fluid accumulation) against stress-relaxing (e.g. cell death and ECM remodelling) processes.
**Solid stress**
The mechanical stress stored within the solid components of a tissue, including cells, their cytoskeleton and the ECM.
**Strain**
A dimensionless measure of deformation that describes the change in size (e.g. length) relative to the original size in response to forces.
**Stress relaxation**
The gradual decrease in stress over time following deformation, usually through fluid redistribution, cell rearrangements or cell death, and/or ECM remodelling.
**Tensile stress**
The internal mechanical force per unit cross-sectional area generated when a biological tissue or cell is subjected to outward-pulling stretching forces.
**Tissue pressure**
The isotropic component of mechanical stress. It is a scalar quantity generated collectively by fluid (hydrostatic) pressure and solid phase mechanical interactions (cell jamming, ECM deformation and tissue confinement). Pressure and compressive stress are equivalent only under isotropic stress states.

The importance of tissue pressure homeostasis is particularly evident in pathological contexts such as cancer, where it has been extensively studied. Uncontrolled tumour growth leads to the progressive buildup of solid stress, as excessive cell proliferation deforms the neighbouring host tissues, the extracellular matrix (ECM) and vascular networks (Stylianopoulos et al., 2012; Nia et al., 2020). Surrounding cancer-associated fibroblasts further remodel this mechanical landscape by exerting compressive stress to drive tumour cell growth (Barbazan et al., 2023; Bertillot et al., 2024), highlighting the importance of mechanical homeostasis in tissue function.

In developmental biology, compared to mechanical cues such as stretch, stiffness and topography, the cellular responses to compressive stress, particularly in three-dimensional (3D) contexts, remain relatively unexplored. This gap is partly due to technical challenges in measuring and manipulating compressive stress in living tissues. Recently, a growing body of research across phylogenetically diverse systems – including mouse, chicken, *Drosophila* and *Arabidopsis*, as well as prokaryotic models (such as *Escherichia coli*) – have revealed that compressive stress can regulate cell proliferation, apoptosis, differentiation and morphogenesis (Biswas et al., 2025; Delarue et al., 2016; Merle et al., 2025; Shyer et al., 2017; Silveira et al., 2025). Hence, there is a need to identify emerging principles and common mechanisms underlying tissue pressure-mediated regulation of development, and to highlight unresolved questions for future studies in this area.

In this Review, we synthesise current advances in understanding the role of tissue pressure in governing development in the plant and animal kingdoms, while evaluating parallel principles in prokaryotes. We first clarify the terminology used to describe tissue pressure and the mechanisms that generate and regulate pressure within tissues. We then discuss examples highlighting the functional consequences of tissue pressure in development, and review evidence that dysregulated pressure homeostasis can lead to developmental defects and ageing. Finally, we propose how advances in experimental and computational approaches for probing tissue pressure in living systems will provide fundamental new insights into cell mechanotransduction and adaptation during development and physiology.

## Defining tissue pressure and compressive stress

In most literature, the terms tissue pressure, compressive stress and solid stress are often used interchangeably. It is therefore helpful to clarify these terms to better understand the broader implications of these parameters in biological contexts. Compressive stress refers to the directional component of mechanical stress that acts to ‘squeeze’ a material along a specific direction. Tissue pressure, on the other hand, is a scalar quantity defined as the average magnitude of isotropic stresses in a living tissue. Tissue pressure and compressive stress are therefore related, and high compressive stress is often associated with elevated tissue pressure. This relationship holds true in symmetric systems where compressive stress is uniformly distributed to create an isotropic stress state, such as in epithelial cysts or tumour spheroids grown within elastic alginate capsules (Alessandri et al., 2013; Di Meglio et al., 2022; Dolega et al., 2017; Helmlinger et al., 1997). In contrast, tissue pressure and compressive stress may differ in systems where compression occurs along a specific direction, such as in the case of *Drosophila* gastrulation (Dey et al., 2025; Vellutini et al., 2025). The relationship between tissue pressure and compressive stress can be better understood in cases where confinement-induced compressive stress leads to a buildup of internal tissue pressure, e.g. during rodent enamel tooth knot formation and *Arabidopsis* shoot regeneration (Shroff et al., 2024; Varapparambath et al., 2022).

Furthermore, compressive stress acting on two-dimensional (2D) or 3D materials can lead to distinct consequences for cell shape, polarity and tissue deformation. For instance, in 2D flat epithelial monolayers, in-plane compressive stress acting parallel to the layer can cause the cells to be elastically compressed, or lead to tissue bending (buckling) out of the plane in order to relieve the stress. In 3D tissues, when stress is isotropic and acts uniformly in all directions, bulk compression can lead to arrested tissue growth, as discussed in later sections. The dimensional context is therefore closely linked to the directionality and mode of stress transmission, which can influence different cellular functions and tissue dynamics.

Classical theories describing tissue morphogenesis were first formulated within continuum mechanics, in which tissues are treated as homogeneous materials that grow and deform collectively rather than as assemblies of individual cells (Taber, 1995). This allows decomposition of tissue deformation into an irreversible growth component and a reversible elastic component (Rodriguez et al., 1994). Such models described how differential growth between neighbouring tissues creates mechanical incompatibilities, generating residual mechanical stress that leads to developmental buckling and folding (Ben Amar and Goriely, 2005). These growth-induced solid stresses were first experimentally demonstrated in confined tumour spheroids, where accumulating solid stress feeds back to suppress proliferation (Helmlinger et al., 1997; Stylianopoulos et al., 2012). In parallel, cell-based descriptions of epithelial mechanics (e.g. vertex models that treat tissues as discrete collections of individual cells) showed how such collective stresses can, in turn, feed back to regulate growth (Shraiman, 2005), offering a complementary, discrete counterpart to the continuum perspective.

Living tissues are often composed of both solid and fluid phases: the solid phase includes cells, the cytoskeleton and ECM components that bear elastic and viscoelastic loads, whereas the fluid phase comprises interstitial fluids or large fluid-filled cavities (lumina) (Lee et al., 2026a). This biphasic organisation allows tissues and cells to behave as poroelastic materials, where the resistance to fluid flow through the porous ECM or the cytoskeleton network drives time-dependent stress relaxation (Moeendarbary et al., 2013; Wang et al., 2020). Together, these poroelastic properties – the hydraulic permeability and ECM elasticity – determine the kinetics of stress accumulation and dissipation, providing a mechanism for the dynamic regulation of tissue mechanics, including tissue pressure. Here, we consider solid stress as the forces transmitted through cellular and ECM structures, distinct from stresses mediated by the fluid phase, such as hydrostatic or osmotic pressure (Chan and Hiiragi, 2020; Jain et al., 2014).

It is also worth noting that tissue pressure is inherently relative. In many biological contexts, differences in tissue pressure between compartments often drive tissue deformation, morphogenesis and associated cellular responses during development.

## Regulators of tissue pressure

Tissue pressure is regulated by active cellular processes and fluid accumulation, as well as by cell non-autonomous factors, such as topological defects and external confinement. Recent studies have highlighted how these mechanisms interact to shape mechanical states across tissues, some of which are discussed below.

### Growth under confinement

**Fig. 1.**
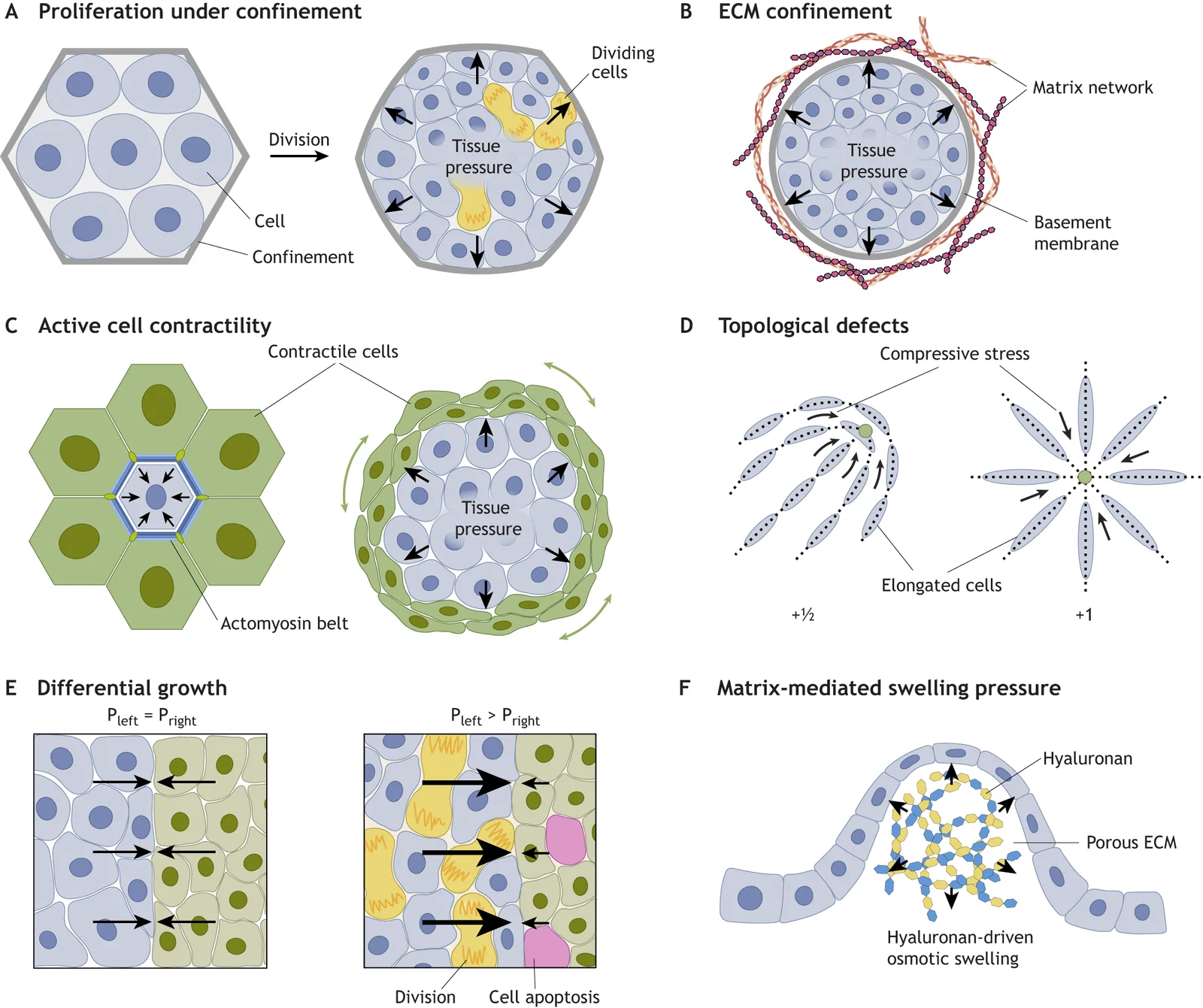
**Emergence of tissue pressure from solid and fluid stress.** (A) Cell proliferation within a confined boundary increases cell density, causing tissue pressure buildup (black arrows) as the tissue attempts to expand further against this confinement. (B) Such external confinement can be imposed by a relatively rigid matrix network or basement membrane that restricts tissue expansion and generates tissue pressure. (C) Actomyosin contractility generates compressive forces at both the cellular and tissue scales. Contractile forces transmitted through intercellular junctions locally compress neighbouring cells (left), while a contractile cell layer surrounding an aggregate of multiple cells acts as a dynamic boundary that compresses the enclosed tissue during active contraction (green double-headed arrows), leading to tissue pressure buildup (right). (D) Sites of localised compressive stress (black arrows) can arise at topological defects with charges of +½ and +1 (which describe the orientation of cells around a defect) within tissues, where discontinuities in cell alignment concentrate forces at a point. (E) Left: adjacent tissues (with identical properties) experience equal tissue pressure (*P_left_* = *P_right_*). Right: tissues with differential growth generates a pressure imbalance, such that the faster growing tissue develops higher pressure than the slower growing tissue (*P_left_* > *P_right_*), driving the displacement of the tissue boundary toward the compartment with lower pressure. (F) Hyaluronan deposition within a porous extracellular matrix (ECM) absorbs water and swells, generating fluid pressure (arrows) that contributes to tissue expansion.

The emergence of solid stress primarily involves cell proliferation within confined environments, a process described as growth-induced pressure (Delarue, 2025). As cells proliferate, the overall tissue volume tends to increase; however, this expansion is often constrained by physical structures, such as neighbouring tissues or the ECM network. When growth is resisted by such semi-rigid boundaries, internally generated forces accumulate, ultimately leading to elevated tissue pressure (Alessandri et al., 2013; Shroff et al., 2024) (Fig. 1A).

The extent of confinement is strongly influenced by the mechanical properties of the surrounding ECM, including specialised ECM structures such as the basement membrane (Fig. 1B). The elasticity and stiffness of these structures typically depend on its composition and crosslinking architecture. For instance, type I collagen, the predominant structural component of the ECM, exhibits pronounced strain stiffening, whereby resistance to deformation increases with applied strain (Nam et al., 2016; Storm et al., 2005; Sun, 2021). Likewise, the basement membrane, which is composed primarily of laminin and type IV collagen, possesses viscoelastic properties that enable time-dependent stress relaxation (Li et al., 2021). As a result, these extracellular structures initially accommodate tissue growth but progressively resist further expansion, thereby promoting pressure buildup within the enclosed, growing tissue. Conversely, ECM and basement membrane remodelling and turnover can partially dissipate accumulated stresses, and such dynamic modulation of pressure buildup is postulated to be crucial for tissue morphogenesis in many organisms, such as *Drosophila* (Diaz-de-la-Loza et al., 2018; Proag et al., 2019; Sui et al., 2018), *C. elegans* (Keeley et al., 2020) and mouse (Harunaga et al., 2014; Kyprianou et al., 2020).

In plants, similar mechanical principles operate through the stiff cellulose cell wall, which constrains volumetric expansion during growth. The cell wall provides structural support and acts as the primary mechanical constraint resisting internal hydrostatic (turgor) pressure (Zhang et al., 2024). Dynamic cell wall remodelling, together with regulated water fluxes, controls stress relaxation and pressure distribution, enabling tissues to relieve or redistribute mechanical stress during key processes, including *Arabidopsis* shoot meristem growth and regeneration (Alonso-Serra et al., 2024; Varapparambath et al., 2022). For example, spatially restricted activation of the cell wall-modifying enzyme XTH9 in cells surrounding progenitor populations alters local mechanical properties, generating mechanical heterogeneity (or stress) between progenitor and neighbouring cells that promotes progenitor expansion and meristem formation (Varapparambath et al., 2022).

### Active cell contractility

Tissues also actively generate pressure through cytoskeletal contractility. Actomyosin networks in animal cells can produce contractile forces that are transmitted across intercellular junctions, allowing local stress generation and propagation across tissues (Campàs et al., 2014) (Fig. 1C, left). Evidence for active generation of compressive stress has been obtained using deformable oil microdroplets (Box 1) embedded in tooth mandible explants, where inhibiting myosin II activity significantly reduces measured compressive stresses (Shroff et al., 2024).

Box 1. Tools to measure and perturb tissue pressure and stress **Stress sensors** – These are categorised into passive and active rheological approaches. In the passive approach, oil microdroplets or deformable beads with known mechanical properties are injected into tissues. Anisotropic stress or isotropic pressure can then be inferred through the shape deformation of the sensor. In active rheology, ferrofluid droplets inserted into living tissues are being deformed through an externally applied magnetic force. The surrounding tissue material properties can then be inferred from the deformation profile of the droplets. Limitations: microinjection of sensors into cells or delicate tissues can be invasive as they physically damage the sample and often alter the local environment, thereby limiting the applications of this technique.

**Micropipette aspiration** – A technique to measure surface tension of a cell or cell aggregate. When applied to multicellular spheroids, according to the Laplace law, P=2γ/R, where P is the spheroid pressure, γ is the surface tension of spheroid and R is the radius of spheroid. Hence the spheroid pressure can be inferred once its surface tension is measured by micropipette aspiration. Limitations: this technique assumes uniform liquid-like properties, which overlooks tissue-level geometric or mechanical heterogeneities.

**Laser ablation** – A method to infer tissue pressure by quantifying the outflow velocity of tissue when it is being cut (ablated) by a focused laser beam. It can also be used to release tissue pressure and study the functional consequence of tissue pressure. Limitations: this approach can only be used for relative pressure comparisons rather than absolute measurements; furthermore, it relies on the assumption that the mechanical properties of a tissue are homogeneous.

**Tissue indentation** – Indentation-based techniques use cantilevers to locally deform cell or tissue surfaces, generating force-displacement curves from which stiffness, viscoelasticity, and pressure can be extracted. Common techniques include atomic force microscopy and nanoindentation. Limitations: this is largely confined to tissue-surface mechanics and can be highly model dependent; furthermore, it relies on assumptions that the material is homogeneous and linearly elastic.

**Stress inference** – A theoretical approach to infer, based on microscopy images, relative cell–cell junctional tension and cytoplasmic pressure in tissues. These models are particularly useful in mapping out epithelial tissue mechanics, where cell junction networks are straight and well defined. Limitations: this technique yields relative values when used alone, and it also relies on assumptions of mechanical equilibrium.

**Osmotic compression** – A method where the addition of high molecular weight dextran molecules into the medium creates a large osmotic stress and induces water efflux from tissues or cell aggregates, effectively exerting global compressive stress on them.

**Gel confinement** – General approach of using 3D hydrogels to generate confinement-induced compressive stress. Under isotropic confinement, this approach also allows the quantification of growth-induced tissue pressure in embryonic tissues and tumour spheroids.

Beyond intrinsic cell contractility, tissues surrounded by contractile cell populations, such as mesenchymal cells, can experience imposed compressive stress and elevated tissue pressure (Fig. 1C, right). For example, contractile theca cells that surround the ovarian follicles exert compressive stress on the internal granulosa cells, maintaining an optimal intrafollicular pressure that promotes follicle growth (Biswas et al., 2025) (see Fig. 3A). Similarly, during tracheal–oesophageal separation, convergent migration of mesenchymal cells generates compressive stresses that constrict the foregut epithelium. Interestingly, when the underlying compressive mesenchymal forces are lost, applying external pressure can restore the tissue architecture (Yan et al., 2025), indicating that the mesenchyme-derived compression is a key driver of foregut morphogenesis. Along similar lines, in developing mammalian hair follicles and avian skin follicles, contractile dermal fibroblasts aggregating around epithelial placodes generate compressive forces, which coordinate proper hair or feather patterning (Shyer et al., 2017; Villeneuve et al., 2024) (Fig. 3F). Collectively, these findings highlight that active contractility – whether generated intrinsically within a tissue or exerted by neighbouring cells – is a major driver of compressive stress and tissue pressure dynamics.

### Defect-induced compressive stress

Many bacterial populations, epithelial and mesenchymal tissues behave as active nematic materials, in which elongated cells align locally to form orientational order (Duclos et al., 2014, 2017; Marchetti et al., 2013; Peng et al., 2016). This alignment becomes increasingly ordered as cell density increases, but continuous, active cellular flows and geometric constraints can distort this orientation field, giving rise to topological defects, which are singular points at which cellular orientation becomes discontinuous (Doostmohammadi et al., 2018; Saw et al., 2018).

Topological defects are classified according to their topological charge, which describes the net rotation of cellular orientation around the defect core (Saw et al., 2018). As cells generate anisotropic contractile forces along their long axes, distortions in cellular orientation near the defect cores lead to local force imbalances. Active nematic theory predicts that such imbalances drive cellular flows and density heterogeneities within tissues (Marchetti et al., 2013). For instance, half-integer defects (when topological charge=±½) are associated with sites of convergent cellular flows that promote local crowding and increased cell density (Kawaguchi et al., 2017; Saw et al., 2017) (Fig. 1D). The buildup of local compressive stress at these defect cores can ultimately trigger cell extrusion from epithelial monolayers (Saw et al., 2017). By contrast, integer defects (topological charge=+1) adopt aster, spiral or vortex configurations that focus active stresses at the defect core, driving convergent flow, local cell accumulation and out-of-plane tissue buckling (Guillamat et al., 2022). Similar to half-integer defects, integer defects in densely cultured myoblasts establish force gradients that concentrate compressive stresses at their cores. However, rather than driving cell extrusion or flow, the resulting stress accumulation at these sites drives localised cell differentiation (Guillamat et al., 2022).

Recently, such integer defects of actin fibres in *Hydra* have been shown to act as organising centres for regeneration (Maroudas-Sacks et al., 2021). Consistent with a developmental role for defect-associated mechanics, externally compressing *Hydra* in stiff agarose perturbs the distribution of topological defects and disrupts body-axis regeneration (Ravichandran et al., 2025).

Collectively, these studies suggest that topological defects can generate local regions of tissue crowding and compressive stress, potentially influencing developmental and regenerative outcomes. Elucidating the downstream consequences of defect-associated stress therefore constitutes an exciting avenue for future research.

### Differential growth and tissue flow

As tissues are mechanically coupled, differences in growth rates or large-scale tissue movements between adjacent cell populations can generate internal mechanical stresses. In most confined systems, proliferating tissues generate increasing compressive stress until a steady state is reached, in which cell division is balanced by apoptosis. When two tissues with different growth rates are mechanically coupled, their interaction is governed by their relative homeostatic pressures. The tissue with the lower homeostatic pressure stops growing and ceases proliferation upon reaching equilibrium. Whereas the neighbouring tissue with a higher homeostatic pressure or higher proliferation rate continues to grow (Basan et al., 2009). Furthermore, this rapidly growing tissue exerts lateral compressive forces on the slower-growing population, pushing it beyond its homeostatic state, and leading to higher apoptosis rates and progressive tissue shrinkage (Fig. 1E). Consequently, mechanical stresses are often distributed non-uniformly across tissues, with faster-growing regions generating compressive stresses in neighbouring slower-growing regions and creating spatial stress gradients that reflect underlying growth heterogeneity.

Several developmental systems illustrate how differential growth contributes to tissue mechanics and morphogenesis. For instance, differential proliferation within the *Drosophila* wing imaginal disc generates spatial patterns of tensile and compressive stresses that feedback to regulate cell division orientation and tissue shape (LeGoff et al., 2013; Mao et al., 2013) (Fig. 3C). Such mechanical stress arising from differential growth can be further amplified during large-scale tissue movements. For example, during *Drosophila* gastrulation, germ band extension generates tissue flows that compress neighbouring regions (Desprat et al., 2008), potentially contributing to the transient folding events that form epithelial invaginations known as the cephalic furrows (Dey et al., 2025; Vellutini et al., 2025).

### Fluid–ECM coupling

In addition to compressive stresses generated through the solid phase of tissues by growth and cellular contractility, fluid pressure and fluid–ECM interactions can also contribute to tissue compression. For example, in plants, fluid storage compartments such as vacuoles can generate turgor pressure through osmotic water influx, producing high internal hydrostatic pressures that are resisted by the surrounding cell wall (Ali et al., 2019). This interaction increases the internal mechanical load within the tissue as the matrix expands against surrounding constraints, contributing to tissue pressure buildup. It is well reported that the hydrostatic pressure in lumen and interstitial fluids can shape development from the mouse embryo to zebrafish semicircular canals, which has been summarised in numerous reviews (Chan and Hiiragi, 2020; Chugh et al., 2022; Navis and Bagnat, 2015; Lee et al., 2026a).

In recent years, emerging evidence has further shown that fluid–ECM coupling also helps orchestrate tissue morphogenesis across diverse animal systems (Fig. 1F). In particular, ECM components such as proteoglycans and glycosaminoglycans can retain large amounts of water, generating osmotic swelling pressures that influence tissue mechanics (Munjal et al., 2021). Notably, hyaluronan forms a highly hydrated, negatively charged network that resists compression due to electrostatic repulsion and water retention, creating a nearly incompressible matrix that can sustain substantial pressure. Interestingly, similar hyaluronan-mediated compressive stresses have also been reported in tumour cells (Voutouri and Stylianopoulos, 2018). Fluid-driven pressure buildup is particularly well characterised in tumours, where elevated interstitial fluid pressure, arising from vascular leakage and impaired lymphatic drainage, contributes to mechanical compression of the surrounding tissue (Jain et al., 2014; Nia et al., 2020). Together, these findings highlight the shared mechanical principles underlying tissue morphogenesis during normal development and tumour growth, where interactions between fluid transport, osmotic swelling and ECM mechanics generate mechanical stresses that shape tissue architecture and morphogenesis.

## Molecular mechanisms sensing compressive stress

Unlike tension, which is directional and often transmitted through focal adhesion complexes, tissue pressure acts isotropically and is distributed across the cells and the surrounding matrix. This makes pressure sensing in 3D environments particularly challenging, as cells experience compression from multiple directions rather than along a single mechanical axis. As a result, identifying discrete ‘sensors’ of pressure has proved challenging. Nevertheless, insights from *in vitro* models, such as 2D epithelial monolayers and tumour spheroids, have begun to reveal how cells may detect and respond to compressive stress (Table 1).

**Table 1.** Mechanical stress measurements across biological systems

Technique	Magnitude of stress or pressure	Reference
Oil microdroplet	Mammary epithelial aggregate anisotropic stress, ∼3.4 kPa	Campàs et al. (2014)
	Rodent incisor anisotropic stress, 0.25-0.4 kPa	Shroff et al. (2024)
	Mouse gastruloid anisotropic stress, 0.2-0.4 kPa	Trani-Bustos et al. (2025 preprint)
Hydrogel deformable bead	Zebrafish embryo tissue pressure, 0.6-1 kPa Tumour colony stress, <1 kPa	Mohagheghian et al. (2018)
		Träber et al. (2019)
Ferrofluid microdroplet	Zebrafish tailbud anisotropic stress, ∼50 Pa	Mongera et al. (2018)
Double emulsion microdroplet	Zebrafish embryo interstitial osmotic pressure, ∼0.7 MPa	Vian et al. (2023)
Atomic force microscopy	Mouse ovarian intrafollicular pressure, 0.1-0.6 kPa	Biswas et al. (2025)
	Onion epidermal cell turgor pressure, 0.1-2.0 MPa	Tsugawa et al. (2022)

### Sensing compression through macromolecular crowding

**Fig. 2.**
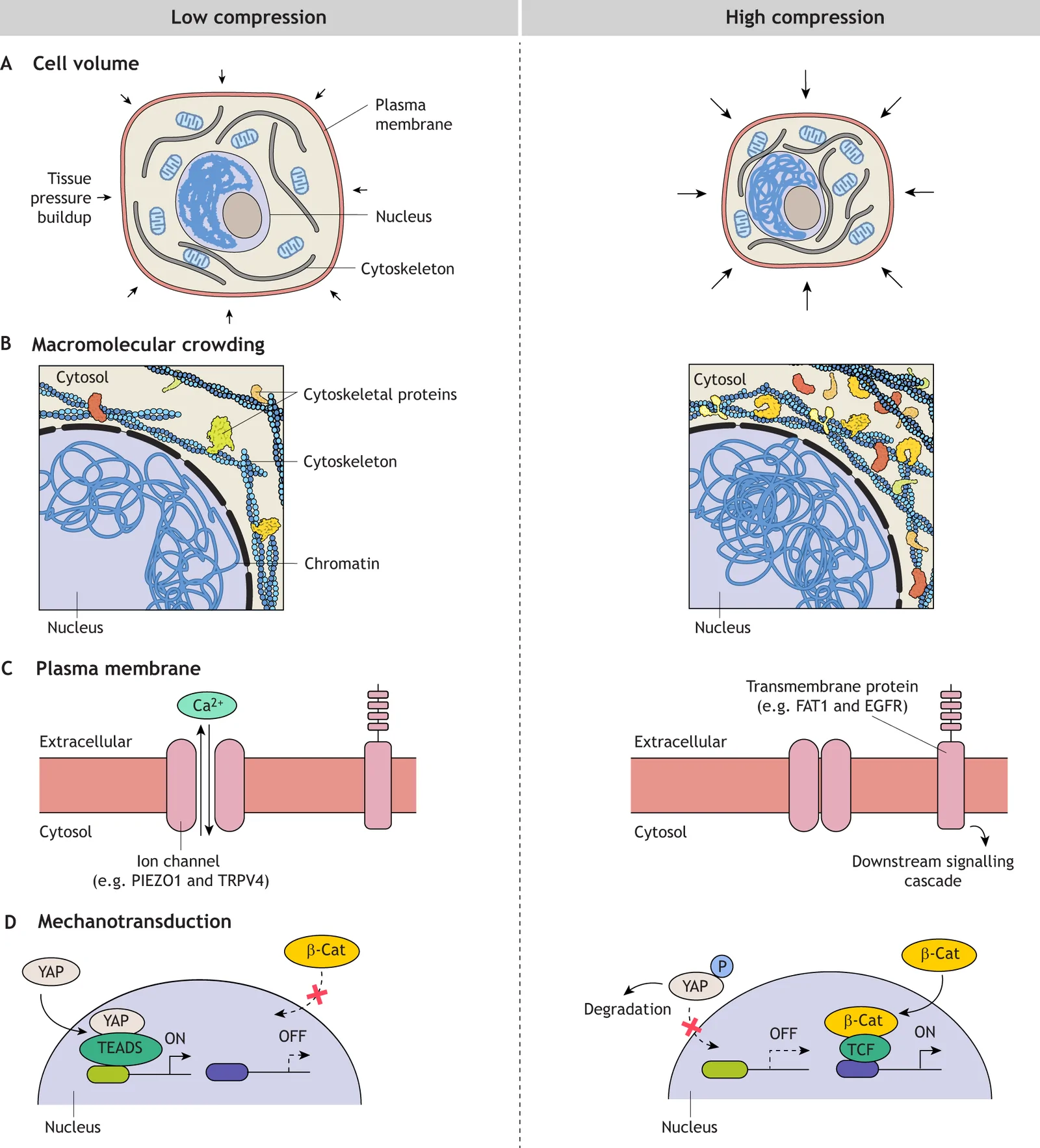
**Cellular responses to compressive stress in mammalian models.** (A-C) Compression reduces cell volume (A) and leads to deformation in cellular components such as the nucleus (B) and plasma membrane (C). Black arrows represent compressive stress. (B) Increased compressive stress enhances macromolecular crowding within the cytosol and nucleus, which may influence biochemical reaction rates and promote chromatin compaction. (C) Compressive stress can modulate the activity of mechanosensitive ion channels (e.g. PIEZO1 and TRPV4) and regulate downstream signalling cascades through transmembrane proteins (e.g. FAT1 and EGFR). (D) Compressive stress may regulate the activity of classic mechanotransduction proteins such as YAP and β-catenin, which leads to changes in the expression of cell cycle and differentiation genes by binding to their respective transcription factors, TEADS and T-cell factor (TCF). P, phosphorylation.

At the cellular level, compression often leads to a reduction in cell volume due to water efflux (Fig. 2A) (Guo et al., 2017). One consequence of this volume reduction is macromolecular crowding, which refers to an increase in intracellular macromolecule concentration within the cytosol (Fig. 2B). In particular, macromolecular crowding has been proposed as a key intracellular mediator, which alters reaction kinetics and activates downstream signalling across all domains of life, including bacteria, archaea, fungi, plants and animals (Alric et al., 2022; Mukherjee et al., 2026). It can either enhance reaction rates due to increased substrate proximity (Jarvis et al., 1990; Kelch et al., 2011) or reduce diffusion rates, thereby hindering molecular transport and slowing diffusion-limited processes (Huang and Ferrell, 2026; Miermont et al., 2013; Nettesheim et al., 2020).

Beyond influencing intracellular biochemistry, compression-induced increases in cellular biomass density can also affect membrane potential in epithelial monolayers, thereby regulating cellular electrophysiology (Mukherjee et al., 2026). In epithelial monolayers, compression-induced increases in biomass density promote membrane hyperpolarisation, potentially because most intracellular macromolecules, including proteins, RNA and DNA, possess a net negative charge at physiological pH. This change in membrane potential subsequently inhibits cell growth and cell cycle progression through activation of the Hippo pathway, which promotes growth-suppressive Hippo pathway effectors and limits proliferative signalling (Mukherjee et al., 2026) (Fig. 2C,D). Together, these findings suggest that cells may sense compressive environments indirectly through changes in intracellular crowding and electrophysiological state, providing a mechanism by which mechanical compression is translated into altered growth and tissue homeostasis.

### Sensing compression through mechanical changes to nuclear architecture

Compression-induced changes in the cell volume can also be transmitted mechanically to the nucleus. Nuclear deformation under compressive stress can alter chromatin accessibility, thereby linking tissue mechanics to gene regulation. In some contexts, chromatin remodelling may also serve as an adaptive response to mechanical stress-induced damage by altering nuclear mechanical properties and protecting genome integrity (Nava et al., 2020) (Fig. 2B). For example, skin epidermal progenitor cells subjected to compression rely on PIEZO1-mediated calcium signalling to reduce heterochromatin formation, and this loss of compact DNA packing leads to nuclear softening (Nava et al., 2020). These findings suggest that under extreme compression, chromatin remodelling can serve as an adaptive response that buffers mechanical forces and helps preserve genome integrity. In contrast, dysfunctional nuclear mechanosensing can lead to nuclear rupture and DNA damage, as frequently observed in cancer cells migrating through confined environments (Denais et al., 2016; Raab et al., 2016).

Similar principles are also observed in the *Arabidopsis* shoot apical meristem, where tissue folding at the boundary between the meristem and emerging organs generates local compressive stresses that deform nuclei (Fal et al., 2021), leading to chromatin compaction and reduced transcriptional activity. Thus, mechanical forces arising from differential growth and tissue geometry can be translated into gene regulatory changes through direct effects on nuclear architecture.

### Sensing compression through ion channels

In parallel with these intracellular responses, compression is also sensed at the plasma membrane. Compressive stress alters plasma membrane tension (across the lipid bilayer), which in turn modulates the activity of mechanosensitive ion channels or other transmembrane proteins, thereby triggering downstream signalling pathways (Merle et al., 2025; Mukherjee et al., 2026; Nam et al., 2019) (Fig. 2C). For example, in confined environments, activation of the stretch-sensitive channel TRPV4 promotes PI3K/Akt signalling, which further inhibits cell cycle progression through the cyclin-dependent kinase inhibitor p27^Kip1^ (Nam et al., 2019). Similarly, other mechanosensitive channels, such as PIEZO1, have been implicated in converting compressive forces into calcium-dependent signalling responses that regulate apoptosis during *Drosophila* leg disc development (Merle et al., 2025), while the EGFR/ERK pathway mediates cell elimination in the *Drosophila* pupal notum (Moreno et al., 2019).

**Fig. 3.**
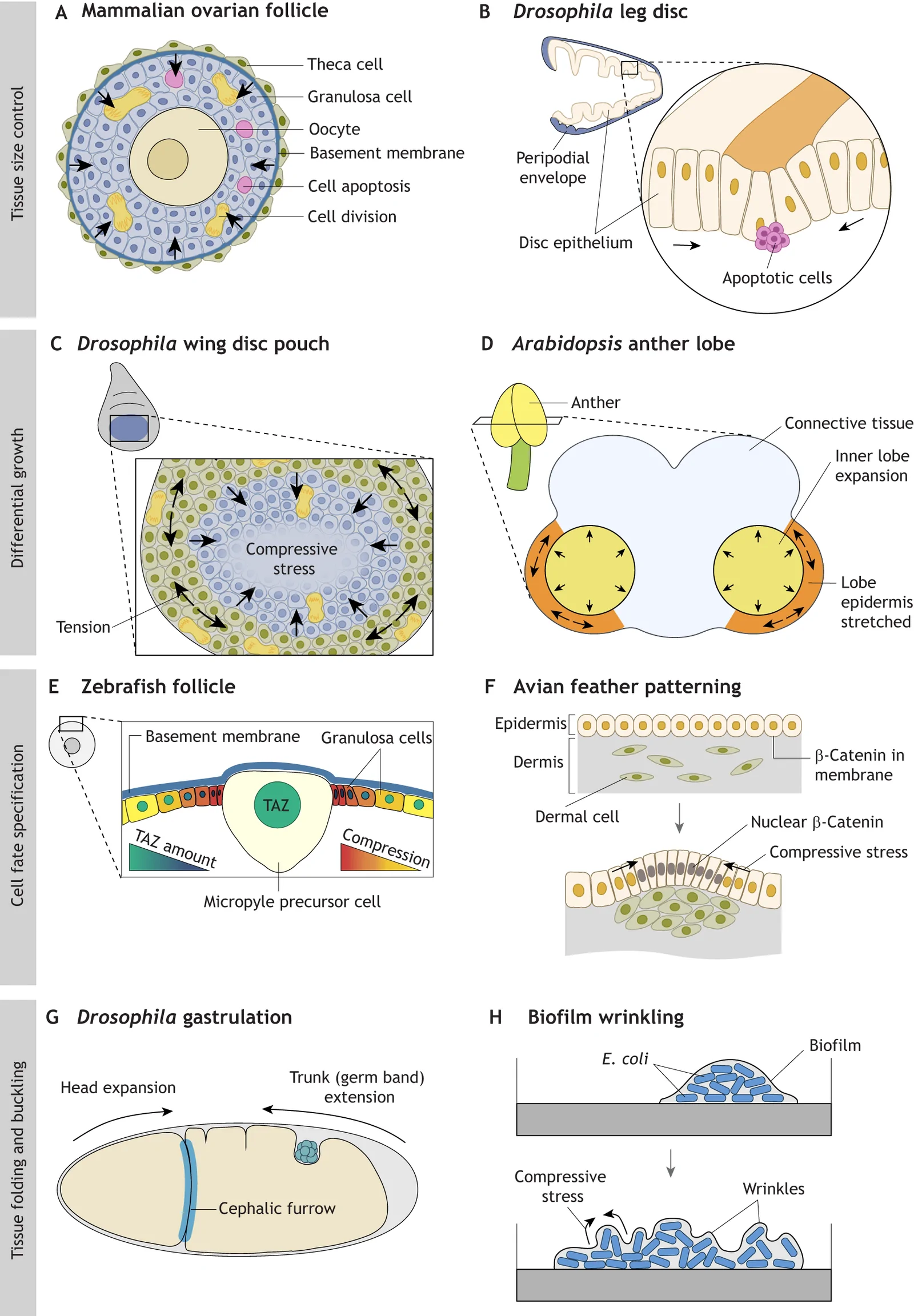
**Modulation of tissue dynamics by compressive stress during development.** (A) Mouse ovarian follicle growth requires contractile theca cell activity and confinement by the extracellular matrix (ECM) to regulate intrafollicular pressure, which, in turn, balances granulosa cell division (yellow) and death (purple). Arrows indicate compressive stress. (B) The *Drosophila* leg disc is mechanically confined by the peripodial envelope, which induces compressive stress buildup (arrows) and eventually cell apoptosis at the distal tip. (C) Differential planar growth in the *Drosophila* wing imaginal disc pouch generates a heterogenous mechanical stress field (arrows), with tensile stresses in the peripheral cells (green) and compressive stress in the central cells (blue). These stresses regulate cell division patterns. (D) During *Arabidopsis* anther lobe formation, faster growth in the inner lobe (single arrows) causes pressure buildup, causing the lobe epidermis to stretch (double arrows). (E) In the zebrafish follicle, rapid expansion of the future micropyle precursor cell mechanically compresses the adjacent granulosa cells, inducing nuclear export of TAZ in the granulosa cells. This restricts micropyle precursor fate to a single cell through mechanical lateral inhibition. (F) In avian skin follicle patterning, dermal cell aggregation generates compressive forces on the overlying epidermis, inducing β-catenin translocation from the membrane to the nucleus and initiating feather follicle fate specification. (G) Cephalic furrow formation during *Drosophila* gastrulation serves as a pressure-relieving transient fold that dissipates excess compressive stress generated from tissue collisions between the head and trunk (germ band) extension. (H) Growth under confinement generates internal compressive stresses in *Escherichia coli* biofilms that drive surface buckling and wrinkle formation.

Collectively, these mechanisms converge on signalling pathways that allow compressive stress to be transmitted to the nucleus, where it can directly influence gene expression and cell fate decisions (Fig. 2D). Canonical mechanotransduction pathways, such as YAP/TAZ signalling, are particularly well studied in this context. Under low levels of compression (e.g. physiological compression during tissue development), YAP/TAZ can translocate to the nucleus and promote cell proliferation through interaction with its transcriptional co-activator TEADS (Aragona et al., 2013; Barbazan et al., 2023; Mukherjee et al., 2026). Conversely, increased compressive stress inhibits cell cycle progression and proliferation in tumour spheroids (Delarue et al., 2014). Other mechanosensitive transcriptional regulators also respond to compressive stress. During avian skin budding, for example, epidermal compression promotes the release and nuclear translocation of β-catenin from cell–cell junctions, where it activates Bmp2 through interaction with its transcriptional partner, T-cell factor, to drive feather follicle formation (Shyer et al., 2017) (Fig. 3F).

## Tissue pressure in developmental regulation

Pressure-induced changes in signalling pathways ultimately translate into changes in gene expression and cell behaviour. As a result, tissue pressure acts as an instructive cue that regulates morphogenesis across diverse systems, by controlling cellular growth dynamics, differentiation and tissue patterning (Delarue, 2025).

### Tissue growth control through cell proliferation and death

Tissue pressure plays a key role in regulating tissue growth through cell density control. Pressure-mediated growth control is hypothesised to ensure robust size homeostasis during development, since mechanical signals can propagate large distances to coordinate growth globally (Lander, 2011; Harmansa and Lecuit, 2021). Early theoretical work proposed that living tissues maintain mechanical homeostasis through bi-directional feedback between growth and pressure (Basan et al., 2009; Shraiman, 2005). In this framework, increased cell proliferation under spatial confinement raises internal pressure, which feeds back to arrest further proliferation or trigger cell death once a critical pressure threshold is met. Pressure homeostasis therefore links cellular behaviours to tissue-scale mechanics, where elevated tissue pressure feeds back to regulate growth rates and cell density.

This mechanical feedback is closely linked to the classical phenomenon of contact inhibition of proliferation, where confluent tissues undergo growth arrest upon reaching a homeostatic mechanical state (Aragona et al., 2013; Di Meglio et al., 2022). Importantly, contact inhibition of proliferation is now understood to arise not only from biochemical signalling, but also from compressive stresses that accumulate as cells become confined within a fixed area (Eisenhoffer et al., 2012). Similar mechanical feedback mechanisms operate in microbial populations, which experience growth-induced pressure since they proliferate in constrained environments, such as biofilms, porous media or host tissues (Delarue, 2025). This self-generated pressure can drive collective phase transitions such as self-driven jamming, a process when the population packs so densely that it leads to a mechanically arrested state with halted spatial expansion (Delarue et al., 2016).

Evidence from mammalian ovarian follicle development also supports a role for pressure in tuning somatic cell density (Fig. 3A). While an optimal level of pressure (around 0.1-0.6 kPa) exerted by outer contractile theca cells is necessary for healthy follicular development, abnormal theca cell contractility perturbs the balance between granulosa cell proliferation and death in mice. These theca cells also assemble a fibronectin scaffold around the follicle, which imposes additional confinement. Similarly, *in vitro* culture of mouse follicles in stiffer matrix confinement significantly reduces the follicle growth rate (Pietroforte and Amargant, 2026). Together, this indicates that tissue confinement forms a negative-feedback loop, whereby the resulting rise in intrafollicular pressure could restrain further follicle expansion. This provides a potential biomechanical mechanism for regulating follicle growth during mammalian development (Biswas et al., 2022).

Beyond inhibiting proliferation, tissues can also respond to excessive accumulation of mechanical stress by removing cells from densely packed epithelia (Eisenhoffer et al., 2012). Mechanically induced apoptosis occurs in crowded epithelial layers and developing tissues, where compressive stress activates mechanosensitive pathways to trigger programmed cell death (Eisenhoffer et al., 2012; Merle et al., 2025). For instance, during *Drosophila* leg disc elongation, compression from the surrounding peripodial envelope induces apoptotic events that contribute to the formation of concentric leg folds (Fig. 3B**)** (Merle et al., 2025). Furthermore, this compression redistributes intracellular mechanical stress, increasing lateral membrane tension and activating PIEZO1. Collectively, these findings demonstrate that tissue pressure functions as a global regulator of tissue homeostasis in terms of coordinating cell density through a balance between proliferation and elimination.

### Differential growth of tissue compartments

Pressure differences arising from differential growth between tissues can drive mechanical instabilities and growth patterns. Recent studies have revealed that this interplay between tissue growth and mechanics is highly conserved across both animal and plant development. In the *Drosophila* larvae wing imaginal disc, tissue deformation and stress measurements revealed a global stress pattern, whereby cells located in the central wing pouch region experience compressive stress, while those at the periphery experience tensile stress (LeGoff et al., 2013; Mao et al., 2013) (Fig. 3C). Along the proximal–distal axis of the wing disc, differential proliferation allows faster-growing regions to generate mechanical forces that stretch neighbouring cells, resulting in cell shape changes that orient the axis of cell division (Mao et al., 2013). Furthermore, theoretical models have shown that differential planar growth rates, combined with ECM compression, drive the initiation of the three conserved apical folds in the wing disc (Tozluoǧlu et al., 2019).

Similarly, during early *Arabidopsis* anther lobe formation, cells in the inner lobe expand more rapidly compared to those in the surrounding epidermis, generating local tension within the epidermis (Silveira et al., 2025) (Fig. 3D). Spatial heterogeneity in growth rates between adjacent regions of the *Arabidopsis* shoot apical meristem likewise generates mechanical stress patterns that influence cell behaviour and organ formation (Hamant et al., 2008; Bauer et al., 2024). This principle is also evident in the *Arabidopsis* root elongation zone, where cells remain laterally confined by surrounding tissues; consequently, growth proceeds under persistent mechanical constraint imposed by neighbouring cells and outer tissue layers (Fujiwara et al., 2023).

This mechanical imbalance – where adjacent cell layers or tissue regions grow at different rates – triggers apoptosis or extrusion of mechanically disadvantaged cells to maintain tissue integrity (Eisenhoffer et al., 2012). This process is closely linked to the concept of cell competition, where ‘loser’ cells are eliminated by ‘fitter’ neighbours (Levayer and Moreno, 2013). For example, in the *Drosophila* pupal notum, the expansion of fast-growing oncogenic clones mechanically compresses neighbouring wild-type cells. This localised crowding suppresses EGFR/ERK signalling and upregulates pro-apoptotic genes such as *Hid*, leading to apoptosis and extrusion of ‘loser’ wild-type cells (Moreno et al., 2019). Ultimately, differential mechanical stress across tissue compartments can function as a quality-control mechanism by regulating cell survival and competition; however, in pathological contexts such as oncogenic transformation, this process can be hijacked to favour the expansion of abnormal cells.

### Cell-fate specification

In addition to regulating tissue growth and homeostasis, compressive stress serves as a crucial mechanical cue for cell-fate decisions, such as differentiation or maturation. During zebrafish follicle maturation, mechanical heterogeneity within the granulosa cell layer (somatic cells surrounding the oocyte) plays a crucial role in the specification of the micropyle precursor cell, which ultimately creates the channel required for sperm entry (Xia et al., 2019) (Fig. 3E). Specifically, a dominant cell within a specialised cluster of granulosa cells accumulates more nuclear TAZ and grows faster than its neighbours. This differential growth generates local compressive stress on adjacent cells, causing them to lose nuclear TAZ and suppressing their growth potential. The cell that retains nuclear TAZ activity eventually adopts the micropyle precursor cell fate.

Similarly, during *Drosophila* germ band extension, compressive stresses in the stomodaeal region trigger the nuclear translocation of β-catenin, which upregulates the transcription factor Twist and specifies the anterior midgut fate (Desprat et al., 2008). Together, these studies highlight β-catenin and TAZ as mechanosensors that link tissue mechanics to eventual cell specification.

Spatial confinement, which can lead to compressive stress buildup as cells proliferate, has emerged as a key regulator of quiescence in many organs, such as the ovary and skeletal muscle. In the ovary, oocytes contained within primordial follicles are usually maintained in a dormant state to preserve the long-term female germline reservoir. Oocyte quiescence is regulated by the FOXO3-AKT pathway, where nuclear localisation of FOXO3 is associated with oocyte dormancy (John et al., 2008; Nagamatsu et al., 2019). Since these primordial follicles reside preferentially in the ovarian cortex, which is a stiffer region compared to the medulla, it has long been suggested that oocyte quiescence could be mechanically regulated by the rigid microenvironment (West et al., 2007; Hornick et al., 2012). Indeed, increasing evidence suggests that oocyte dormancy is reinforced by a mechanically constraining microenvironment, such as the collagen-rich niches in the mouse ovarian cortex (Nagamatsu et al., 2019, 2026). When this mechanical constraint is relieved through ECM degradation, FOXO3 translocates from the nucleus to the cytosol, triggering oocyte activation (Nagamatsu et al., 2019). Recent work further showed that this mechanotransduction pathway is mediated through KIT receptor tyrosine kinase signalling. Under external pressure, the KIT receptor on the oocyte membrane is internalised and sequestered into cytoplasmic vesicles in a dynein-dependent manner. This receptor sequestration reduces oocyte sensitivity to the KIT-PI3K-AKT signalling cascade, thereby preventing FOXO3 nuclear export and maintaining oocyte quiescence (Nagamatsu et al., 2026).

In mammalian skeletal muscles, muscle stem cells (also known as satellite cells) are maintained in a quiescent state within a highly confined environment between the myofibre and the basement membrane. Upon muscle injury, muscle stem cells experience a loss of apical compression from the damaged myofibres, causing them to enter the cell cycle to support regeneration (Yin et al., 2013). Interestingly, applying artificial apical compression to activated muscle stem cell surfaces is sufficient to revert them to a quiescent state, likely through increased expression of Notch downstream genes (Tao et al., 2023). Together, these findings highlight how tissue mechanics directly can act as an instructive regulator of cellular state, serving as a primary physical cue that helps maintain quiescence or permits activation in response to changes in the cellular environment.

### Tissue folding and buckling

Beyond regulating growth, tissue pressure contributes directly to morphogenesis by driving large-scale tissue deformations. During growth under confinement, when compressive stress accumulates unevenly or exceeds the threshold for mechanical stability, tissues can undergo buckling, folding or invagination to relieve mechanical stress (Di Meglio et al., 2022). Such deformations not only dissipate accumulated stress but can also be harnessed to generate tissue architecture during development.

During *Drosophila* gastrulation, collisions between actively proliferating and moving tissues generate localised compressive stress that drives formation of the cephalic furrow (Dey et al., 2025) (Fig. 3G). The cephalic furrow subsequently acts as a mechanical buffer, absorbing and redistributing excessive stresses generated by germ band extension and mitosis at the head–trunk boundary, thereby contributing to epithelial stability (Vellutini et al., 2025). More broadly, epithelial sheets subjected to compressive stress can bend or invaginate, generating folds that contribute to organ shaping during development, including gastrulation and avian follicle patterning (Nelson et al., 2005; Shyer et al., 2017).

Beyond epithelial folding, pressure-driven tissue deformation can also contribute to morphogenesis in other systems. For example, simple metazoans such as *Hydra* exhibit remarkable plasticity in regeneration, where applying external compression induces topological defects within the supracellular actin network, ultimately promoting the development of viable two-headed animals (Ravichandran et al., 2025). This demonstrates how pressure-induced reorganisation of the cytoskeleton can reshape body axis formation during regeneration.

Brain folding provides another striking example of how compressive stresses can generate tissue architecture. Theoretical models first proposed that cortical folding arises through a buckling instability, whereby rapid growth of the outer cortical layer is constrained by the slower expansion of the inner layers (Nie et al., 2010; Richman et al., 1975). This mechanism was later recapitulated in synthetic brain organoids, where differential growth between the outer and inner layers produces wrinkling patterns resembling those of the cerebral cortex (Tallinen et al., 2014, 2016). Furthermore, regional variations in tissue stiffness or growth rates can bias where compressive stresses accumulate, thereby influencing the orientation and positioning of cortical folds. For instance, addition of ECM proteins, including HAPLN1, lumican and collagen I, to human foetal neocortex explants increased tissue stiffness that eventually caused cortical folding (Long et al., 2018). Since increased ECM stiffness also promotes local cortical progenitor division (Fietz et al., 2010), we hypothesise that it may generate local cell crowding and compressive stress that are subsequently relieved through buckling, although this mechanism remains to be tested.

The ability of compression to generate large-scale shape changes is not limited to multicellular, eukaryotic tissues. Mechanical compression and growth-induced stresses also alter cell shape and colony morphology in bacterial populations. For example, the accumulation of internal pressure, which generates solid-like behaviour in densely packed microbial populations, can result in mechanical instabilities and wrinkle-like deformations (Delarue et al., 2016) (Fig. 3H). Similarly, localised cell death within a biofilm can redistribute mechanical stresses, leading to vertical deformation of the colony (Asally et al., 2012). Furthermore, self-induced mechanical stress arising from growth under confinement can also trigger biofilm formation in *E. coli* (Chu et al., 2018).

Together, these examples illustrate how compressive stresses can be relieved through tissue-scale (or colony-scale) morphological features, ranging from epithelial invaginations and cortical folds to bacterial wrinkling. In many cases, these mechanically driven shape changes are not simply passive consequences of growth, but actively contribute to tissue organisation and morphogenesis.

## Dysregulated tissue pressure in development and ageing

When tissues experience abnormal tissue pressure, it often manifests into uncontrolled tumour growth, developmental disorders and age-associated organ dysfunction. In this section, we highlight key evidence illustrating how altered cell mechanics and dysregulated ECM remodelling can drive these pathological outcomes.

### Developmental disorders

Proper morphogenesis requires the precise spatial and temporal regulation of tissue pressure over time, and disruption of this balance can lead to developmental disorders. As mentioned previously, quantification of tissue-scale stresses remains limited in most developmental systems (Table 1) and, consequently, ‘pressure thresholds’ to determine insufficient or excessive pressure leading to impaired morphogenesis have not been established. When pressure generation is insufficient, tissues may fail to produce the mechanical forces required for large-scale deformation. For example, lumen expansion relies on controlled accumulation of hydrostatic pressure that promotes the inner cell mass lineage specification during mouse blastocyst formation (Chan et al., 2019; Ryan et al., 2019).

Conversely, excessive pressure may restrict cell dynamics and alter signalling pathways. For instance, *in vitro* models of asthma and bronchoconstriction, which utilise primary human bronchial epithelial cells, showed that excessive compression of the airway epithelium sheet by the surrounding bronchial smooth muscle promotes tissue jamming, causing the tissues to transition into solid-like states (Park et al., 2015, 2016). Compression of the airway epithelium is not only a passive consequence of tissue mechanics, but can actively remodel the surrounding microenvironment. Compressed epithelial cells promote airway smooth muscle proliferation and contractility during bronchoconstriction, creating a feedback loop that further elevates compressive stress (Park et al., 2016). Together, these findings reveal that abnormal tissue pressure is detrimental to development, highlighting that successful morphogenesis requires maintaining pressure homeostasis within an optimal range.

### Ageing

Ageing tissues exhibit altered mechanical properties, including increased stiffness, changes in ECM composition and impaired stress relaxation (Kohn et al., 2015). While these alterations likely influence tissue pressure regulation, the underlying mechanistic relationship remains poorly characterised. In the cardiovascular system, a key hallmark of ageing is increased fibrosis, which is characterised by excessive ECM deposition and reduced matrix turnover (Biernacka and Frangogiannis, 2011; Horn and Trafford, 2016; Selman and Pardo, 2021). Fibrosis is largely driven by the activation of fibroblasts into contractile myofibroblasts, which tend to secrete abundant ECM components (Frangogiannis, 2021). This often creates a confined microenvironment that amplifies the interstitial pressure around cells. The activation of cardiac fibroblasts leads to ECM remodelling, increased collagen deposition and progressive tissue stiffening (Kurose, 2021). This combination of increased matrix stiffness and pressure overload drive maladaptive remodelling, that involves cardiomyocyte hypertrophy, apoptosis and impaired contractility (Pandey et al., 2018). Fibrotic stiffening further restricts the ability of cardiac tissues to relax during diastole, reducing stress dissipation and promoting sustained mechanical compression at the tissue level (Zile et al., 2015). These findings suggest that age-related ECM remodelling in the heart not only alters stiffness but also perturbs pressure homeostasis, contributing to progressive functional decline.

Similar principles are emerging in reproductive ageing, particularly in the ovary, where ECM mechanics play a crucial role in regulating follicle development. Early work established that ovarian ageing is accompanied by progressive fibrosis and collagen accumulation, leading to a mechanically restrictive microenvironment that disrupts follicle growth and ovulation dynamics (Mara et al., 2020). Age-associated increases in the ovarian stromal ECM drive tissue stiffening, which has been linked to impaired follicle development, reduced ovulation efficiency and increased follicle death (Umehara et al., 2022; Pietroforte and Amargant, 2026). We speculate that excessive ECM stiffness may elevate intrafollicular pressure by restricting follicle expansion during growth. Supporting this biophysical mechanism, *in vitro* studies using cultured mouse secondary follicles in 3D alginate hydrogels revealed that follicle development is highly sensitive to mechanical confinement, with stiffer matrices restricting growth and reducing developmental competence by triggering fibro-inflammatory pathways (Pietroforte and Amargant, 2026). Conversely, relieving compressive constraints through mild digestion of collagen in aged mouse ovaries can restore follicle health and boost ovulation (Lin et al., 2026; Umehara et al., 2022). Furthermore, aged mouse ovaries also exhibit reduced phosphorylated myosin light chain in theca cells, suggesting diminished contractile activity, which likely disrupts pressure homeostasis inside aged follicles required for coordinated growth and oocyte maturation (Biswas et al., 2025).

Together, these findings suggest that age-associated ECM remodelling contributes to increased tissue stiffness, thereby altering the generation, transmission and dissipation of tissue mechanical stress. This likely shifts the tissues away from a state of pressure homeostasis towards chronic mechanical imbalance. While direct measurements of tissue pressure in aged tissues remain limited, the studies discussed above provide strong evidence that dysregulated tissue pressure is an important yet underappreciated contributor to age-related functional decline of organs.

## Conclusions and future directions

Dissecting the mechanosensing pathways requires precise characterisation and manipulation of tissue pressure *in vivo.* Unlike many of the tools developed for 2D systems (Sugimura et al., 2016), where forces can be easily applied and measured, probing mechanics in three dimensions remains a significant challenge (Box 1). Emerging techniques, such as deformable stress sensors, allow assessment of 3D tissue-scale mechanical stress *in vitro* (Dolega et al., 2017) and *in vivo* (Campàs et al., 2014; Kim et al., 2024; Mongera et al., 2018; Träber et al., 2019) (Table 1). Tissue-scale laser ablation is also increasingly used to measure internal tissue pressure. By tracking the rate of tissue outflow at the ablated site, this technique estimates the underlying compressive forces within the tissue. This approach has been widely applied to study how compressive stress regulates tissue growth in tumour, ovarian follicles and mammalian gastruloids (Barbazan et al., 2023; Biswas et al., 2025; Trani-Bustos et al., 2025 preprint).

Since most of the above-mentioned approaches are rather invasive to biological cells and tissues, researchers have developed less destructive, complementary approaches to infer tissue pressure. For spherical cell aggregates, tissue pressure can be calculated using the Laplace Law, which dictates that internal pressure is directly proportional to surface tension and inversely proportional to tissue size (indicated by the radius of curvature). By measuring surface tension independently, often via micropipette aspiration, researchers can further determine the internal pressure of the spheroid (Yousafzai et al., 2022). Alternatively, nano- or micro-indentation techniques are increasingly used to measure hydrostatic pressure in plant cells (Beauzamy et al., 2015; Tsugawa et al., 2022) and growing tissue pressure in avian embryos (Chan et al., 2023). Finally, image-based stress inference methods based on cell–cell junction force balance also allow estimation of both junctional tensions and intracellular pressures across tissues (Roffay et al., 2021; Ichbiah et al., 2023; Sugimura and Ishihara, 2013; Kong et al., 2019).

Experimental perturbations offer distinct ways to study tissue mechanics. While methods such as laser ablation can release tissue pressure, applying osmotic compression using large macromolecules drives an increase tissue pressure (Delarue et al., 2014; Dolega et al., 2017, 2021; Monnier et al., 2016). This approach has provided new insights into pressure-induced development of pancreatic organoids, mouse embryos and ovarian follicles (Shroff et al., 2024; Biswas et al., 2025; Lee et al., 2026b).

In recent years, organoid systems have emerged as a powerful platform bridging *in vitro* and *in vivo* studies (Zhao et al., 2022). Organoids are increasingly able to recapitulate key aspects of tissue architecture, including lumen formation, epithelial organisation and ECM confinement, while allowing better control of mechanical parameters. For example, 3D gel confinement studies have shown that tissue pressure and curvature regulate the cell cycle in epithelial cysts (Di Meglio et al., 2022). While recent work demonstrated the instructive role of growth-induced pressure on guiding gastruloid elongation (Trani-Bustos et al., 2025 preprint), further research is required to understand how biophysical cues, such as tissue pressure, integrate with biochemical signalling to drive robust organoid self-organisation (Weichselberger et al., 2026).

Looking forward, overcoming current experimental challenges will require stronger integration with theoretical models, which will enable prediction of pressure distributions in systems where direct measurement is not feasible. A range of cell-based modelling approaches, including the vertex model, Voronoi model, Cellular Potts model and granular or deformable cell models (Barton et al., 2017; Bi et al., 2016; Hirashima et al., 2017; Runser et al., 2024), provide useful strategies to simulate multicellular dynamics, allowing *in silico* exploration of how tissue pressure emerges and evolves over time. Notably, recent work incorporating the poroelastic nature of tissues (Dolega et al., 2021; Recho et al., 2019) shows that pressure is not solely a consequence of solid stress accumulation, but emerges from the interplay between cell division, viscous dissipation and fluid permeation (Recho et al., 2019). However, predicting tissue pressure changes across development remains challenging because confining structures, such as the surrounding matrix network, actively remodel rather than acting as static boundaries. To address this, future predictive models must couple dynamic compressive stresses with solid–fluid biphasic growth dynamics to accurately reflect *in vivo* microenvironments.

An emerging and largely unexplored frontier is the role of pressure as an evolutionarily conserved driver of development across life forms. Recent studies have shown that mechanical compression can trigger multicellular, tissue-like organisation in archaea, indicating that pressure-driven structural transitions may pre-date the evolution of complex multicellularity (Rados et al., 2025). It is also likely that organisms inhabiting chronic, high-pressure environments have evolved specific adaptations to survive. For example, deep-sea comb jellies adapt to extreme hydrostatic pressure by enriching their membranes with specific phospholipids (e.g. plasmenyl phosphatidylethanolamine) that exhibit spontaneous negative curvature. At sea level (low pressure), these phospholipids naturally form an unstable membrane. However, under the crushing weight of the deep ocean, this unique membrane composition compresses perfectly into a stable and fully functional bilayer (Winnikoff et al., 2024). Together, these observations raise the possibility that tissue pressure is not only a regulator of development in canonical animal systems, but also a conserved physical mechanism that has been co-opted across domains of life to shape multicellular organisation under chronic and intense mechanical stress.

Overall, tissue pressure is emerging as a central regulator of multicellular organisation in development, homeostasis and disease. However, many questions remain regarding how cells sense and respond to compressive stress and reciprocally modify tissue pressure. Since most of these processes rely on multiscale feedback mechanisms, decoding the fundamental principles of tissue mechanics and addressing related challenges require a truly multi-disciplinary approach, combining advanced experimental tools with predictive modelling.
